# Bellevue Hospital College

**Published:** 1861-05

**Authors:** 


BELLEVUE HOSPITAL COLLEGE.—This is the title of a new medical school recently chartered by the New York Legislature, in connection with the Bellevue Hospital, on the grounds of which the college buildings will be erected. The names of the gentlemen composing the faculty have not as yet been announced; but we believe that it is their intention to prolong the course, and to make clinical teaching at the bedside the important feature in their plan of study.

